# Perceptions of counsellors and youth-serving professionals about sexual and reproductive health services for adolescents in Soweto, South Africa

**DOI:** 10.1186/s12978-018-0455-1

**Published:** 2018-02-06

**Authors:** Mamakiri Mulaudzi, Busisiwe Nkala Dlamini, Jenny Coetzee, Kathleen Sikkema, Glenda Gray, Janan Janine Dietrich

**Affiliations:** 1 Perinatal HIV Research Unit (PHRU), Faculty of Health Sciences, University of the Witwatersrand, Chris Hani Road, Diepkloof, Soweto, Johannesburg, 1864 South Africa; 2 South African Medical Research Council, Cape Town, South Africa; 3 Duke University, Department of Psychology and Neuroscience, Durham, USA

**Keywords:** Adolescent-friendly, Youth-friendly, HIV, Sexual and reproductive health, HIV counselling and testing (HCT), Qualitative

## Abstract

**Background:**

Adolescents in South Africa remain vulnerable to HIV. Therefore, it is crucial to provide accessible adolescent-friendly HIV prevention interventions that are sensitive to their needs. This study aimed to investigate the perceptions of HIV counsellors and other youth-serving professionals about the barriers to providing adolescent youth-friendly sexual and reproductive health services to adolescents in Soweto, South Africa. The study also explored how sexual and reproductive health services in South Africa could be improved to become more accessible to adolescents.

**Methods:**

The research team conducted two focus group discussions with HIV counsellors, and 19 semi-structured interviews with youth-serving professionals from organisations working with adolescents. Audio-recorded data were transcribed verbatim and analysed using thematic analysis.

**Results:**

The results of the study reveal that counsellors were expected to give adolescents HIV counselling and testing (HCT) but felt restricted by what they perceived as inflexible standard operating procedures. Counsellors reported inadequate training to address adolescent psychosocial issues during HCT. Healthcare provider attitudes were perceived as a barrier to adolescents using sexual and reproductive health services. Participants strongly recommended augmenting adolescent sexual and reproductive health services to include counsellors and adolescents in developing age- and context-specific HIV prevention services for adolescents.

**Conclusion:**

Continuous upskilling of HIV counsellors is a critical step in providing adolescent-friendly services. Input from all relevant stakeholders, including counsellors and adolescents, is essential in designing adolescent-friendly services.

## Plain English summary

This study reports on the experiences of HIV counsellors and other youth-serving professionals in providing adolescent-friendly sexual and reproductive health services for adolescents in Soweto, South Africa. Information was collected using focus group discussions with HIV counsellors and face-to-face individual interviews with youth-serving professionals from different organisations in Soweto. The study investigated perceptions of HIV counsellors and other youth-serving professionals about the barriers to providing sexual and reproductive health services for adolescents in Soweto and how these services can be improved to become more adolescent-friendly. The results reveal a need to involve key stakeholders in the design and implementation of healthcare services for adolescents. Findings from this study will add to a deeper understanding of the challenges and barriers to providing adolescent-friendly sexual and reproductive health services to adolescents in Soweto, South Africa.

## Background

Approximately 7.1% of young South Africans aged 15–24 are infected with HIV, with only 14.3% receiving antiretroviral (ARV) treatment, the lowest proportion of treatment exposure when compared with other age groups [1]. At an adolescent-friendly HIV counselling and testing (HCT) centre in Soweto, 4% of young people aged 15–24 were HIV infected in 2015 [2]. Prior studies in South Africa have reported high rates of unintended adolescent pregnancies [3, 4]. In 2012, 3.9% of pregnant adolescent girls under 15 years and 19.3% of young women aged 15–24 years, were HIV-positive [5].

Young people in South Africa continue to face barriers when accessing sexual and reproductive health services [6–10]. Commonly cited barriers include lack of confidentiality and privacy, long waiting times (often with adults from the same community), inconvenient operating hours, the remote location of clinics, and a fear of parents finding out about clinic visits [8–10]. Service provider attitudes have been widely reported as hindering adolescents’ access to health services, and studies indicate that adolescents tend to avoid health facilities because of the unfriendly, judgmental attitudes of healthcare workers [8, 9]. Researchers found that even after providers received training on how to respond to adolescents seeking sexual and reproductive health services, some remained biased and judgmental [8].

Increased access to and acceptability of sexual and reproductive healthcare among adolescents, achieved through adolescent-friendly services, are a priority for the South African Department of Health (DoH) [9]. Adolescent-friendly services refers to services that are prompt, geographically accessible and welcoming, and that can assure adolescents of confidentiality. These services are staffed with trained personnel to address the sexual and reproductive needs of adolescents [9]. In 1999, the South African DoH, in partnership with loveLife (a sexual health programme for adolescents), established the National Adolescent-Friendly Clinics Initiative (NAFCI). Managed by loveLife between 1999 and 2006, NAFCI was later incorporated into the South African DoH Adolescent and Youth Friendly Services programme [10], an intervention aimed at establishing standards for the provision of adolescent-friendly services in the public health sector [9]. The three key objectives of the NAFCI approach were: to provide high-quality adolescent reproductive and healthcare services and to improve access to health services for adolescents; to establish adolescent-friendly standards and criteria across national healthcare clinics in South Africa; and to equip healthcare workers with appropriate skills for adolescent-friendly service provision [9, 11]. Research on sexual and reproductive health services offered to adolescents rarely addresses service providers’ experiences and perceptions. Therefore, this study investigated counsellors’ and youth-serving professionals’ perceptions of sexual and reproductive health services for adolescents to better understand how services can improve to become adolescent-friendly.

## Methods

We conducted a qualitative cross-sectional study to explore the experiences of HIV counsellors and other youth-serving professionals in providing sexual and reproductive health services for adolescents. The aim was to explore barriers to providing adolescent-friendly sexual and reproductive health services.

### Study setting

The study was conducted at the Perinatal HIV Research Unit (PHRU), located at Chris Hani Baragwanath Hospital in Soweto, South Africa. Soweto is the most populated black urban area in the city of Johannesburg, Gauteng province, with an estimated population of 2 million people [12].

### Study design and participants

A qualitative study was conducted using focus group discussions (FGDs) and semi-structured interviews (SSIs). The FGDs were conducted with HIV counsellors to obtain diverse perspectives in a group setting. As HIV counselling and testing (HCT) is regarded as a non-sensitive topic, the group setting allowed for sharing of work practices and experiences of HCT with adolescents. Participants in the FGDs were purposively selected because they provided HCT services to adolescents, and were recruited from programmes and research sites based in hospitals, clinics and NGOs.

Semi-structured interviews (SSIs) were conducted to obtain specific and in-depth information from professionals working with adolescents. Seven Soweto-based organisations, including research sites and programmes providing services to adolescents, were purposively identified to recruit youth-serving professionals. The SSI participants comprised multidisciplinary team members providing services to both adults and adolescents.

### Ethics

Study procedures were approved by the institutional review boards at the University of the Witwatersrand, Johannesburg, South Africa and Duke University, Durham in the United States. Permission for participation was obtained from managers at HCT centres in Soweto. HIV counsellors and youth-serving professionals gave informed consent for participating in the study and for audio-recording the FGDs and SSIs. Anonymity of participants was retained and audio-recordings used participant identity numbers. Participants received a ZAR50 (~ $7) reimbursement for participating in the study.

### Data collection

#### Focus group discussions

Trained facilitators conducted the focus group discussions (FGDs) using a semi-structured interview guide. Each FGD hosted 8–12 participants, comprising HIV counsellors only, and lasted an average of 2 h including refreshment breaks. Interviews were conducted in private rooms at the PHRU. The FGD guide consisted of open-ended questions, with additional probing questions to elicit further information regarding counselling services for adolescents. Discussions elicited information on successful and unsuccessful adolescent sexual and reproductive health service strategies, challenges in adolescent counselling, and recommendations for improving adolescent sexual and reproductive health services.

#### Semi-structured interviews

Semi-structured interviews (SSIs) comprised youth-serving professionals, including HIV counsellors, who were not part of the FGDs. A trained interviewer administered the semi-structured interview guide, which consisted of open-ended questions with additional probing questions. The interview guide was designed in collaboration with a paediatrician, a psychologist, a nurse, social scientists and social workers, all of whom worked with the adolescent population. The SSI guide was designed to elicit in-depth information about participants’ perceptions and beliefs about adolescents’ sexual behaviour and HIV risk. In addition, participants were asked for suggestions to improve sexual and reproductive health services for adolescents.

#### Analysis

The FGDs and SSIs were audio-recorded, transcribed verbatim, translated into English, and entered into Maxqda for data analysis [13]. Transcripts were read by two researchers to facilitate data immersion. Data were first analysed thematically by identifying themes via coded text [14], and the development of themes and codes was data-driven. The primary analyst reviewed the first two transcripts line by line to gain an overall understanding of the data to develop codes, whereafter a second analyst developed a codebook. A meeting including a senior researcher was organised with both analysts to discuss the codebook and to check for consistency. Text from the transcribed data were coded and organised according to themes. Finally, major themes were defined in relation to the aims of the study, to identify key issues regarding perceptions about adolescent sexual and reproductive health services. The researchers arranged a meeting with participants to discuss the findings.

## Results

### Demographic characteristics of HIV counsellors and other youth-serving professionals

Two mixed gender FGDs (FGD1, *n* = 10; FGD2, *n* = 12) were carried out with 22 HIV counsellors. Two participants were unable to participate because of a scheduling conflict. In total, 10 male and 12 female HIV counsellors, were aged 19–45 years, from similar ethnic and cultural backgrounds (Zulu and Tswana were the two dominant ethnic groups in Soweto) took part. Work experience as counsellors ranged from one to 8 years. All but one participant (with a degree in counselling) had received basic HIV lay counselling training from a variety of organisations.

Semi-structured interviews were conducted with 19 professionals who had an adolescent caseload (*n* = 9 males and 10 females): two counsellors, two doctors, two nurses, three HIV project coordinators, two community outreach officers, two peer educators, two pastors, one teacher, one community liaison officer and two social workers. For inclusion in the study, professionals had to have worked with adolescents in Soweto for at least 1 year. Participants were aged 23–43 and were Tswana- or Zulu-speakers, except for one English-speaking participant. The study staff obtained permission from the organisations to conduct interviews, whereafter candidates were invited to participate. Three teachers and one social worker refused to participate (Table 1).

**Table 1:** Demographic characteristics

Participant characteristics	Frequency (*n* = 41)	Percentage (%)
Gender		
F	22	54
M	19	46
Age (range, years)		
19–25	20	49
26–32	9	22
33–39	9	22
40–46	3	7
Title/Occupation		
Community Liaison Officer	1	2
Doctor	2	5
HIV Counsellor	24	59
Outreach Officer	2	5
Peer Educator	2	5
Professional Nurse	2	5
Project Coordinator	3	7
Social Worker	2	5
Teacher	1	2
Pastor	2	5
Work Experience (range, years)		
1–5	29	71
6–10	10	24
10–15	2	5
Type of Organisation		
Church	2	5
Community centre	2	5
Hospital	5	12
NGO	21	51
Research site	9	22
Schools	2	5

Key themes identified were: barriers to provision of adolescent-friendly services, and revisiting adolescent sexual and reproductive health services.

### Barriers to provision of adolescent-friendly sexual and reproductive health services

Barriers to provision of adolescent-friendly sexual and reproductive health services included provider attitude and clinical environment in health care clinics, restrictive standard operating procedures, lack of consultation with relevant stakeholders, and insufficient training in adolescent HCT.

#### Provider attitude and clinical environment in public health care clinics

Discussions with participants from FGDs and SSIs revealed a critical gap between sexual and reproductive health service provision and adolescent needs. They addressed, primarily, provider attitudes and the clinical environment as barriers to adolescents’ access to healthcare. Participants had reservations about integrating adolescent-friendly services into long-established public healthcare clinic settings. HIV counsellors reported that although public clinics were being improved to provide reproductive health services for adolescents, the clinic environment and the healthcare workers were not adolescent-friendly. ‘*They [legislators] are busy with the development of a friendly environment for adolescents at the clinics but I doubt that it will work. I am saying this because of the attitude from the clinics, because adolescents don’t go to the clinics, really they don’t. The environment at the clinics – the health workers, like most of them, they are judgmental; most health workers are not friendly to youth.*’ (P2 Male counsellor, FGD2)

*‘A lot of them [adolescents], they know about teenage pregnancies and they have to go to a clinic where it’s not teenage friendly and when they get there, they are told they are too young to have sex.*’ (Male Study Coordinator, SSI 5)

In FGD2, a male counsellor said that ‘*adolescents don’t go to the clinics, really they don’t. Why I am saying this? First, the attitude from the clinics- when they need [the] morning after pill, they go to the chemist not at the clinics. Even the condoms they don’t fetch them from the clinics, they buy them at the pharmacy.’*

Two nurses who participated in the study had different opinions and reported that customer care among clinics and healthcare workers was improving and changes were taking place to accommodate adolescents. One nurse said, ‘*I see that they enjoy coming here because now it’s not like before. Hence we see most of them; they are using the facility especially family planning”.* (Female nurse, SSI 15).

However, perceptions of provider attitudes towards adolescents appeared to be inconsistent. During an SSI, a nurse stated, *‘There are mean nurses but there are good nurses[too]… It’s unfortunate that the South African public, it’s like every time when they go to the clinic they meet the mean nurses only. They never get to meet the good nurses.’* (Female clinical nurse, SSI 4).

Another nurse shared her experience in providing sexual and reproductive health services to female adolescents, stating, ‘*In this clinic I tell them that you are taking advantage, you are now coming here every day.’* (Female nurse, SSI 15).

#### Restrictive standard operating procedures

Some HIV counsellors who participated in this study worked in organisations that engaged in research on adolescent HIV prevention and treatment. Standard operating procedures (SOPs) may be developed using different counselling models and differ across organisations, but all give instructions to counsellors on how they should conduct their counselling sessions to ensure consistent quality service provision. HIV counsellors, who worked in HIV prevention research for adolescents, reported dissatisfaction with the SOP’s guiding counselling procedures. They stated that the inflexibility of SOPs restricted provision of adolescent-friendly services. One of the counsellors said that ‘*what annoys me the most in counselling is the fact that you are supposed to make this change and yet there is this protocol that says do not [do this] do not [do that].’* (P5 female counsellor, FGD2).

Another counsellor maintained that *‘this SOP thing that you mustn’t be sitting like this or like that, it doesn’t matter, but whatever [SOP] law that is set, let it be just a free environment. When you get there, feel free; if you want to put your feet on the table, if it means that person will feel comfortable, let it be, if it’s going to make them open …’* (P2 male counsellor, FGD2).

Standard operating procedures included strict behavioural standards to which counsellors were expected to conform, which counsellors perceived as making the environment too rigid and uncomfortable for an adolescent client base. While there was disagreement about how SOPs had to be applied in the counselling session, counsellors recognised that SOPs were merely guidelines. One counsellor stated: ‘*You have to understand that the manual [SOP] is just there to guide you.’ (P3 female counsellor, FGD1).*

Similarly, participants in the key informant interviews commented that counsellors should not have to adhere strictly to the counselling manual. One counsellor said: *‘I don’t entirely believe that everything must be according to what this manual requires…’* (male counsellor, SSI 3).

#### Lack of consultation with stakeholders

Participants from both the FGDs and SSIs raised concern over the lack of consultation with service providers and adolescents in developing SOPs. In their view, while they were expected to provide appropriate counselling services, there was a lack of consultation with the relevant people who knew what was taking place in counselling sessions. For example, one counsellor stated: *‘It’s one thing for a company to say this is what counsellors might do, but not be in the one-on-one session where counsellors are. It might not always be practical’.* (P2 male counsellor, FGD2).

A female counsellor from FGD2 shared her perception regarding the perceived lack of consultation with counsellors when developing adolescent HCT guidelines: ‘*It is not realistic for me, for directors, to sit and decide what counsellors should say….’*

Semi-structured interview participants commented on the lack of consultation with adolescents. A male project manager shared his perception of the lack of consultation with adolescents when developing interventions for them: ‘*What we do, we sit in boardroom(s) like this and just think that we know what they [adolescents] are thinking outside there ... It won’t have that impact. I need to go down there to these young people on the grassroots level and find out what they want.’*

Participants advised that interventions or programmes would not have the desired effect if adolescents were not involved in their design and implementation. They recommended that adolescents be involved in the process of designing or planning from the beginning, including designing HCT programmes:

*‘… Involve teenagers in the planning and whatever protocols*.’ (Male peer educator, SSI 6)

*‘Include teenagers in the programmes. I think that would make a major, major difference.’* (P5 female counsellor, FGD2)

A male physician suggested that adolescents provide input about the HIV testing process, regarding how they would like it to be done: *‘We can ask kids how they would prefer HIV testing to be done.’* (Male physician, SSI 10).

#### Insufficient training in adolescent HCT

In addition to SOP restrictions in providing adolescent-friendly HCT, participants’ perceptions and experiences drew attention to the limited counselling training that they received. Counsellors in this study stated that they had received limited or no training in counselling adolescents. While all counsellors had general HIV/AIDS counselling skills, only a few had received formal training in adolescent development.

*‘The training that we get is completely directed at how to deal with children. I also got training last year on child participation and even there it deals with how you deal with children.’ (P1 male counsellor, FGD1)*

While some counsellors had received training specifically geared for adolescent counselling, the length of training was generally brief. This ineffective and low-quality training gave rise to concerns directed at beneficiaries of the services. A male peer educator (SSI participant) suggested that *‘the duration for the training must not be too short. It must be longer because we are dealing with the lives of other people; you can’t give someone wrong information’.*

Although counsellors were expected to provide adolescent-friendly HCT, they had experienced cases that required in-depth counselling knowledge, including rape and psychosocial issues. One counsellor stated, *‘We don’t only focus on HIV/AIDS ...; we deal with rape, suicide, everything affecting teenagers.’* (P5 female counsellor, FGD1).

Despite evidence suggesting the complexities of providing adolescent HCT, accounts suggested that counsellors felt ill-equipped to manage those cases: *‘Let’s say you are just an HIV counsellor, then they must take you for training on psychosocial counselling. That means if you do happen to pick up, for example, that ‘the problem is that they don’t love me at home that’s why I sleep around’ then you can deal with it.’* (P5 female counsellor, FGD2).

Other counsellors recognised that adolescence was a unique stage of development, with complex issues that may be challenging for counsellors with limited counselling skills. Counsellors highlighted the need for further training in circumventing or reducing the number of referrals to external organisations. The limited training that many counsellors received resulted in a system of referrals to social workers or other professionals to ensure that clients received support. Some of the counsellors were dissatisfied because they had to refer cases that were perceived to be beyond their counselling level of competence and experience. *‘The problem is that they tell you that if you come across this you will have to refer to the social worker and now you have started, your client understands you more and [has] built that trust, but now if you refer him to the social worker, it is going to be different and I am telling [you] he might not come back.’* (P2 male counsellor, FGD2).

### Revisiting sexual and reproductive health services for adolescents

Participants expressed the need for improvement in adolescent sexual and reproductive health services. They recommended the implementation of healthcare service provision characterised by a prompt, entertaining and welcoming environment that would encourage adolescents to talk freely. They alluded to the need to correct attitudes and provide sexual and reproductive health services at the adolescent’s level of understanding, with an innovative approach to HIV information provision. ‘*The approach is very important, you know, and like creating a very friendly atmosphere where the adolescent can feel safe to say whatever that they want to say.’* (Female clinical nurse, SSI 4)

*‘They really hate waiting … they hate sitting on those benches for hours. They want something that’s quick, if you can get a place where they come in and out.’* (P1 Male counsellor, FGD1)

Participants highlighted the need to tailor adolescent health services to be culturally relevant and age specific. A peer educator from the SSIs said that ‘*when you are talking about adolescents you have to break it in this way: the background, and then the social norms and the communities … An adolescent from Soweto will be different from an adolescent from the suburbs. You have to know what their values are so that you can understand the adolescent individually.’* (Male peer educator, SSI 6).

Participants agreed that effective healthcare services had to be designed to be age- and language-specific, while being delivered at a developmentally appropriate level. *‘We need to be able to understand what is their thinking, what are they going through, what makes them tick, what excites them and we bring our interventions at that level.’* (Male peer educator, SSI 8).

Participants reported that adolescents were experiencing HIV/AIDS information fatigue, which made it difficult to keep them interested in HIV education programmes. They emphasised that adolescents were well informed about HIV/AIDS and that programmes on HIV education were perceived as boring, hence the call for innovative programmes. *‘Most of the youth, they rather do something else than come here and listen to someone talking [about HIV/AIDS]. They will say … [they] have heard it before; it’s boring.’* (Female nurse, SSI 15).

## Discussion

Our study aimed to capture the experiences of youth-serving professionals and counsellors who work with adolescents, and who experience the systems and challenges that service providers and adolescents encounter in accessing and providing sexual and reproductive health services. Our findings indicate that HIV counsellors were generally prepared to provide adolescent-friendly services to the adolescent client base. However, this enthusiasm was curtailed by their perceptions that HIV counsellors were inadequately trained and were not included in the design of adolescent HCT interventions.

The South Africa government has adopted and initiated training on adolescent HCT based on the WHO and the Centre for Disease Control and Prevention guidelines [15]. Access to HCT in South Africa has increased, with more than 4500 public health clinics across the country providing voluntary counselling and testing and provider-initiated counselling and testing [15]. Access to HCT by adolescents in South Africa increased from 8.7% in 2008 to 51.6% in 2012 among females and from 19.9% in 2008 to 37.3% in 2012 among males [1]. However, due to the burden of HIV in South Africa, HCT remains the responsibility of lay counsellors [16] with limited and generalised skills who, are less confident in providing HCT to adolescents and young key populations [17].

In our study, HIV counsellors considered training on normal adolescent development and management of psychosocial issues, together with practical experience, as essential to enabling counsellors to provide services beyond basic HCT. Although these psychosocial issues were presented during HCT provision, they were beyond the scope of HCT. While the WHO recommendations for additional training to provide adolescent-specific services is echoed by physicians [18, 19] and counsellors [17, 20], addressing psychosocial issues during HCT is beyond the scope of lay HIV counsellors’ ability. The notion of HIV counsellors came about as a means of reducing the strain on healthcare workers, in which they would provide pre-and post-test counselling to prepare clients for HIV testing. At the same time, HCT is a critical entry point for other services for adolescents. This paper argues that widening the scope of practice for HIV counsellors would require them to undergo extensive training and education and would require changing the criteria for the selection of HIV counsellors. The training needs reported by counsellors in this study are similar to those reported in previous studies in South Africa [17, 21]. Similarly, Coyle and Soodin [21] found that counsellors identified the need to expand HIV counselling training to include skills to address a range of issues beyond HIV/AIDS, including suicidal ideation, alcoholism and relationship problems [21]. Mwisongo et al. [17], reported that lay counsellors in their study reported a lack of standardised counselling and testing training, and a need for counselling skills for specific groups such as discordant couples, homosexuals, older clients and children.

There appeared to be confusion among participants around the regulatory aspects of the counselling SOP and how it informed the counselling session. In this context, the SOP is a document that outlines procedures to be followed when conducting HCT. The SOP offers essential step-by-step guidelines and is usually developed to facilitate consistency in the application of procedures, reducing the chances of miscommunication and ensuring the safety of the clients or patients [22]. An SOP can serve as a document for counsellors to maintain high quality service provision for adolescents and links with key objective 1 of the National Adolescent-Friendly Clinics Initiative (NAFCI) to provide accessible and acceptable health services for adolescents [9]. Some participants reported being restricted by their SOPs and desired greater leeway to tailor their sessions to individual adolescents. Including essential personnel such as counsellors in the development of SOPs for HCT could address the experiential recommendations of counsellors more adequately. Particularly striking about this finding was that counsellors stated a disconnect between SOPs and providing adolescent-friendly services owing to the restrictive nature of SOPs. To our knowledge, this is a unique finding in our study, but it echoes previous research findings that reported the need to engage all relevant stakeholders from programme design through to implementation [23, 24]. Engaging counsellors in developing SOPs may enhance understanding of the importance of SOPs and how they can best be implemented to meet the needs of all stakeholders.

According to the South African HCT policy guidelines, adolescents are a unique group and are at increased risk of HIV [15]. As a vulnerable group, adolescents are faced with particular risks for HIV including early sexual debut, unprotected sex, peer pressure, the need to belong, sexual coercion and gender-based violence [15], and HCT services have to be tailored to address adolescent-specific needs. In particular, youth-serving professionals should receive training that provides a sound understanding of adolescent-friendly approaches, adolescent development and appropriate medical, psychosocial and developmental options according to age and maturity [15]. Generally in South Africa, training on HCT is provided by different accredited HCT organisations [17] and rarely focuses on understanding adolescent development. Overall, the findings reveal that sexual and reproductive health services, including HCT, for adolescents in Soweto, South Africa is occurring, but that providers require specific professional training to deliver consistent adolescent-friendly services.

Participants in this study reported that relevant stakeholders, particularly counsellors, were not involved in designing HIV interventions for adolescents, and recommended their involvement in future developments. Study participants recommended further that adolescents, the recipients of the service, should also be included in designing an HIV intervention for adolescents. The *Save the Children* project is a positive example of an adolescent-friendly approach involving adolescents, parents and teachers in improving adolescent-friendly sexual and reproductive health services at pharmacies [25]. In their study, the demand and use of the services increased for both male and female adolescents, with adolescents returning for repeat services. Moreover, training of pharmacists to provide adolescent-friendly services had a positive effect on the pharmacists as well as their service provision. Adolescents were comfortable with the services and experienced less age discrimination from pharmacists.

Another critical idea that emerged in this study was counsellors’ lack of confidence in referring adolescents for psychosocial support when required. Although not ascertained in this study, underlying factors such as lack of understanding of the concept of referral [26] and power relations [27] whereby counsellors feel responsible for the life of the adolescent may give rise to counsellors’ reluctance to refer adolescents. Other actors may include the lack of evidence for effective referral pathways and comprehensive healthcare service provision. Several studies have shown that adolescents are unlikely to transition to further services at a different location. Moreover, adolescents may be referred to services that are not adolescent-friendly due to limited availability of comprehensive adolescent-friendly healthcare services in South Africa. Adolescents should, ideally, be able to access a variety of services at one clinic. This may decrease the number of cases requiring referral, improve client retention and may be beneficial to adolescent clients. Where comprehensive service provision is impossible, adolescent clients should be referred to services that provide appropriate, and accessible support.

## Limitations

The results reflect the experiences and perceptions of the participants, and may not be representative of all HIV counsellors and youth-serving professionals in Soweto. This paper is limited in that it does not include adolescent perspectives. Lastly, FGDs as a qualitative method, have been criticised for facilitating opinions that may be focused on the group norm. Despite the limitations, our study obtained perspectives from relevant contributors about sexual and reproductive health services, including HCT for adolescents in Soweto.

## Conclusion

Continuous training and upskilling of lay counsellors and youth-serving professionals is a critical step in providing adolescent-friendly services. Additionally, all relevant stakeholders, particularly counsellors and adolescents, must be involved in designing adolescent-friendly services.

## Abbreviations

FGDs: Focus group discussions
HCT: HIV counselling and testing
NAFCI: National Adolescent-Friendly Clinic Initiative
SOPs: Standard operating procedures
SSIs: Semi-structured interviews
